# Child-oriented and partner-oriented perfectionism explain different aspects of family difficulties

**DOI:** 10.1371/journal.pone.0236870

**Published:** 2020-08-19

**Authors:** Konrad Piotrowski

**Affiliations:** Institute of Psychology, SWPS University of Social Sciences and Humanities, Poznań, Poland; Unviersity of Sheffield, UNITED KINGDOM

## Abstract

The aim of the study was to verify the relationship between child-oriented and partner-oriented perfectionism, and their associations with narcissism and with difficulties in the romantic and parental domains. A total of 459 individuals participated, 264 women and 195 men (*M*age = 33.88, *SD* = 4.39). Child-oriented perfectionism and partner-oriented perfectionism were related to each other and positively correlated with narcissism. Partner-oriented perfectionism turned out to be a specific predictor of difficulties in the romantic relations, whereas child-oriented perfectionism was found to be a predictor of difficulties in parental relation. The results suggest that studies on other-oriented perfectionism should take into consideration concrete individuals at whom perfectionistic expectations are directed (e.g. partner and children). This will enable a more precise investigation of the influence of perfectionism on family life and a better understanding of the social consequences of other-oriented perfectionism.

## Introduction

Perfectionism is defined as having extremely high standards of performance with an accompanying tendency to exhibit critical self-evaluation and concerns about being negatively evaluated by others [1, 2]. Most scholars also agree that the development of perfectionism is strongly rooted in relationships with caregivers in childhood and adolescence characterized by pressure, unrealistic expectations, and critique [1, 2]. Hewitt and Flett [2] distinguished three specific facets of perfectionism that have both intra- and interpersonal aspects: *Self-oriented perfectionism* is defined as having perfectionistic expectations of oneself; *socially prescribed perfectionism* is a belief that other people, especially close ones, expect perfection from an individual; and *other-oriented perfectionism* is expecting perfection from other people. Previous studies proved that these different aspects of perfectionism are positively related to each other, but also have their own specificity [2–4].

Researchers studying different facets of perfectionism tend to initially overlook other-oriented perfectionism [5], focusing more on the expectations directed toward oneself. However, in recent years there has been a significant increase in the interest in studies on the other-oriented aspect of perfectionism [5–7]. People who expect perfection from others differ from those who expect perfection mainly from themselves. Individuals who require perfection from themselves are characterized by conscientiousness, achievement motivation, and a fear of making mistakes and negative evaluation [for a review see 8]. In contrast, other-oriented perfectionists are more narcissistic, Machiavellian, psychopathic, less agreeable, and oriented towards domination, control, and emphasizing their own power and superiority [3, 4]. In their case, perfectionistic motives and behaviors are oriented to a smaller extent towards the achievement of internalized standards of their own functioning, and more towards regulating their unstable self-esteem, which is typical of narcissistic individuals. For this reason, other-oriented perfectionism is considered to be the core characteristic of narcissistic perfectionism [6, 7, 9]. For narcissistic perfectionists, it is important to control others and be superior over them. As Smith, Sherry, and Saklofske [5] describe them, ‘they experience other people as annoyingly defective—and their self-appointed job is to fix others’ mistakes’ (p. 268). In order to achieve this, they exert pressure, instigate conflicts, humiliate, and react with anger to shortcomings of their close ones [5]. At the same time, trait narcissism (a personality trait characterized by narcissistic grandiosity and narcissistic vulnerability [10]) and trait perfectionism are specific predictors of adjustment, which is why measuring both of these constructs in studies is justifiable [11, 12].

In most studies on other-oriented perfectionism, no specific individuals towards whom unrealistic expectations are formulated are usually distinguished [2]. Researchers seek rather the causes and effects of the general tendency of perfectionists to set high expectations for other people [3, 4], only differentiating sometimes between the domains to which these high expectations pertain (e.g. individuals in the professional environment or at home without, however, specifying particular people in these environments). In turn, studies on perfectionistic expectations towards concrete individuals, which enable a more specific investigation of the influence of perfectionism on social interactions, have so far been limited to romantic partner-oriented perfectionism, which is referred to as ‘dyadic perfectionism’ [13]. The aim of the current study was to broaden this perspective by including the aspect of perfectionism directed at one’s children, i.e. child-oriented perfectionism. While studies on partner-oriented perfectionism have been carried out in the past [13–18], the present study is the first to analyze specifically child-oriented perfectionism from the perspective of the parents. The issue of perfectionism oriented toward children has been present in the studies on perfectionism since the beginning, being studied, usually retrospectively, as a potential early cause of perfectionism in an individual. Researchers study whether perfectionists’ caregivers had excessively high expectations toward them [1] or how strong current concerns of perfectionists about others’ expectations are [2]. These approaches confirmed that high expectations and a critical attitude toward a child or a teenager are an important correlate of the development of perfectionism [19]. However, in those studies either perfectionists themselves describe their caregivers and their perfectionistic parenting [19] or the caregivers’ perfectionism is directly studied as a general personality trait [20]. No previous studies have been conducted on perfectionistic expectations oriented specifically towards children from the perspective of the parent themself. Thus, we do not know whether child-oriented perfectionism is a specific form of other-oriented perfectionism. Answering this question could help us better understand perfectionistic parents and allow for a more detailed understanding of their role in the children’s development. The present study was planned as a first step in assessing the usefulness of child-oriented perfectionism for studying family difficulties experienced by parents.

The aim was to verify whether child-oriented perfectionism and partner-oriented perfectionism are related to each other, and whether they are related in a similar way to narcissism. Also, verification was sought as to whether partner-oriented perfectionism and child-oriented perfectionism constitute specific predictors of difficulties experienced by parents in their romantic and in their parental relationships.

### Domain-specific other-oriented perfectionism

Many researchers claim that perfectionism, both self-oriented and other-oriented, can manifest itself more in certain specific domains than in others [21–23]. The studies on domain-specific other-oriented perfectionism conducted so far suggest that people may have domains in which this aspect of perfectionism is more salient. Other-oriented perfectionism is analyzed in these studies, though, as a general attitude towards other people who belong to various domains of life. Mitchelson and Burns [24] investigated 67 mothers and found that other-oriented perfectionism tended to be more visible in the workplace (e.g. *I have high expectations of the people who are important to me at work)* than at home (e.g., *I have high expectations of the people who are important to me at home)*, yet other-oriented perfectionism at home still correlated with higher parental distress. In turn, Dunn, Dunn, and McDonald [25] observed that young athletes were characterized by a higher level of general other-oriented perfectionism in the sports domain (e.g. *In sport I have high expectations of the people who are important to me*) than in the educational domain (e.g. *In school I have high expectations of the people who are important to me*). Although such studies deepen significantly our knowledge about the role of other-oriented perfectionism, they also have serious limitations. They focus on various domains (e.g. sport, work, family), and not on concrete individuals with whom perfectionists interact in these domains. This does not enable us to look into the dynamics of specific relations that the perfectionist creates with significant others, because in the work environment, for example, the perfectionist encounters many different people: managers, subordinates, colleagues, etc. In fact, the same applies to every other domain in which the perfectionist enters into social interactions, including the domain of family life in which we have a number of different individuals: a partner, children, own parents, siblings. In order to fully understand the role of other-oriented perfectionism, we have to adopt a more specific approach, one that focuses on specific individuals towards whom perfectionistic expectations are formulated. This approach is found in studies on partner-oriented or dyadic perfectionism. Research into partner-oriented perfectionism suggests that a strong expectation of perfection from a partner is connected with lower satisfaction from the relationship, lower engagement in relations and a lower propensity to forgive the partner his/her mistakes [14, 16, 18]. However, Shea, Slaney, and Rice [13] postulate that partner-oriented perfectionism should be considered with reference to three different dimensions: *High Standards* (setting high expectations for a partner, expecting perfection from the partner), *Order* (expecting that a partner will be well-organized and ordered), and *Discrepancy* (the perception that a partner consistently fails to meet the high standards that one has set for him/her). The main assumption of this approach is that people formulate specific, sometimes very high, expectations of their partners (High Standards and Order) and monitor the degree of their implementation (Discrepancy). Studies based on this multidimensional approach indicate that the three dimensions of partner-oriented perfectionism proposed by Shea, Slaney, and Rice [13] are indeed characterized by a distinct specificity, suggesting that the one-dimensional approach has only limited application, because it turns out that it is not the setting of high standards for the partner in itself that causes conflicts and low satisfaction with the relationship, but rather the high perceived degree of discrepancy between his/her high standards and the perceived degree of their attainment by the partner. Therefore, it is not only a matter of what these expectations are, but whether the partner, in the eyes of the perfectionist, lives up to them. This is why the dimension of *Discrepancy* is the main predictor of difficulties in a romantic relation, whereas *High Standards* and *Order*, when controlling for *Discrepancy*, can even be positively connected with the quality of the relationship [13, 15, 17], which suggests that these two dimensions belong to the adaptive aspects of perfectionism [26].

To measure these three dimensions of partner-oriented perfectionism, the Dyadic Almost Perfect Scale (DAPS [13]) was developed. The approach proposed by Shea, Slaney, and Rice [13] was also applied in the present study, however it was extended to the parental dyad, because it was assumed that in the case of children such an attitude is also possible. Parents can formulate perfectionistic expectations towards their children (high standards and order) and monitor the degree of their attainment (discrepancy), similarly to in the romantic domain. We can suppose also that the effects of perfectionism on the parental relation are similar to those observed in the case of the romantic relationship. If the child does not meet the parents’ high expectations (higher Discrepancy), it can lead to an increase in parental stress, a lack of satisfaction with parenthood, and feeling burdened by this role. This prediction remains in line with the results of a recent study conducted by Piotrowski [27], in which it turned out that the higher the mother’s general other-oriented perfectionism, the stronger is her conviction that becoming a parent was a mistake. As Piotrowski suggests, the mother’s unrealistic expectation that her child will meet her high standards can constitute a source of strong tensions and frustration and, as a result, it can cause disappointment with the parental role.

### Research problem and hypotheses

The study was dedicated to the investigation of the specificity of other-oriented perfectionism in the context of a relation with a partner (partner-oriented perfectionism) and with a child (child-oriented perfectionism). Due to the lack of questionnaires enabling a multidimensional measurement of child-oriented perfectionism, one of the prerequisites of the study became the preparation of a questionnaire for measuring this facet of other-oriented perfectionism. For this purpose, the items from the Dyadic Almost Perfect Scale (DAPS [13]) were modified so that they enabled the measurement of child-oriented perfectionism. This scale was called the *Children Dyadic Almost Perfect Scale* (C-DAPS).

The following hypotheses were tested in the study:

1. It was predicted that the dimensions of partner-oriented perfectionism (High Standards, Order, and Discrepancy) would be positively, but not strongly, correlated with the dimensions of child-oriented perfectionism, and that partner- and child-oriented perfectionism would be linked to a higher level of narcissism. Such results could suggest that child-oriented perfectionism can have similar roots to partner-oriented perfectionism but also possess its own specificity.
2. On the basis of past studies on domain-specific other-oriented perfectionism [24, 25], it was predicted that there could occur differences in respect of levels of High Standards, Order, and Discrepancy regarding the partner and the child (within-subject comparisons). Due to the lack of earlier studies on child-oriented perfectionism, it was difficult to formulate an unequivocal hypothesis about the direction of these differences. Therefore, this hypothesis was treated as an area for exploration.
3. It was also predicted that the discrepancy between expectations and the degree of their attainment by the child would be a specific predictor of difficulties in parenthood, even when controlling for narcissism [see 11] and partner-oriented perfectionism. Analogically, it was assumed that the discrepancy between expectations and the degree of their fulfillment by the partner would be a better predictor of subjectively perceived problems in the sphere of the intimate relationship. Confirmation of this hypothesis would support the thesis about the legitimacy of measuring child-oriented perfectionism and the thesis about the person specificity of other-oriented perfectionism [25]. In the case of High Standards and Order, it was predicted that by controlling for Discrepancy, they would not be associated with difficulties in the romantic and the parental domain, or that they would be negatively linked to them, confirming the thesis about the potentially adaptive function of perfectionistic strivings [26].

## Materials and methods

The study was reviewed and approved by the Departmental Ethics Committee, University of Social Sciences and Humanities, Poznań, Poland (decision number 180901). The participants provided their written informed consent to participate in this study.

### Participants and procedure

Before the study was conducted a power analysis was performed with the use of G*Power 3.1.9.4 software [28] in order to determine the minimum required sample size for a linear regression analysis with about 10 predictors that was expected to be performed (narcissism, the three dimensions of the DAPS, the three dimensions of the C-DAPS, and the potential controlled variables). Smith et al. [7] reported in their meta-analysis that a mean effect size for a relationship between other-oriented perfectionism and narcissism is from .16 (for narcissistic vulnerability) to .31 (for narcissistic grandiosity), and Stoeber [18] found similar effects for the relationship between partner-oriented perfectionism and relationship satisfaction. Based on these observations, it was calculated that about 100 participants were needed to detect an effect size of .30, with alpha level of .05 and a power of .95.

The survey was conducted online. There was only one measurement point in this cross-sectional study. In order to reach participants, the researcher availed himself of his private contacts and the snowball method. The participants were informed about the aim of the research and signed a consent form to participate. The inclusion criteria were having at least one child older than three, being currently in an intimate relationship (it was not verified whether the partner was the mother/father of the child), and being no more than 40 years old (a maximum age limit was used as it would provide the opportunity to study people who were still involved in their children’s lives to a significant extent).

A total of 515 individuals participated, however, 56 of them were excluded as they did not finish the survey or were older than 40. Eventually, 459 individuals (264 women and 195 men), between the age of 21 and 40 (*M* = 33.88, *SD* = 4.39), were included in the final sample. The investigated individuals had from one (34.2%) to four (1.5%) children. The youngest children were 6 months old, while the oldest were 21 years of age, but the vast majority of the investigated parents had children in the preschool and early elementary school age (mode = 3.0 years, median = 5.0 years, *M* = 5.89, *SD* = 3.29). The mean age at which the participants had become parents for the first time was *M =* 26.55, *SD* = 4.03 (median = 27.0, mode = 28.0), which is close to mean values in the Polish population. Some 78% of the participants were married, whereas 22% were in informal relationships. The mean duration of romantic relationships was *M* = 9.56 years, median = 9.0, *SD* = 4.61. The financial position of the investigated individuals was quite good: 46.4% of the participants stated that *they do not have any financial problems and their financial situation is good*, 49.7% reported that *they sometimes have some financial problems*, *but their financial situation is quite average*, and only 3.9% of the subjects stated that *they have serious financial problems and that their financial situation is bad*.

### Measures

#### Narcissism

In order to measure narcissism, the *Super-Brief Pathological Narcissism Inventory* [SB-PNI; 29; Polish adaptation by Rutkowska and Prusik, unpublished] was applied. The scale is composed of 12 items, and is a part of the full version of the PNI [10]. The scale enables the attainment of a global pathological narcissism score, in the scope of which there are aspects of both grandiose narcissism (e.g. *I often fantasize about being recognized for my accomplishments*) and vulnerable narcissism (e.g. *Sometimes I avoid people because I’m concerned that they’ll disappoint me*). An investigated individual gives answers on a scale ranging from 0 (*not at all like me*) to 5 (*very much like me*). The reliability of the scale was .88.

#### Partner-oriented perfectionism

To measure partner-oriented perfectionism, the *Dyadic Almost Perfect Scale* (DAPS; 13) was used. The scale consists of 26 items that create three scales: High Standards (6 items, e.g., *I expect my partner to try to do her/his best at everything she/he does*), Order (4 items, e.g., *I expect my significant other to be an orderly person*), Discrepancy (16 items, e.g., *My partner’s best never seems to be good enough for me*). The questionnaire items are evaluated on a seven-point scale ranging from 1-*strongly disagree*, to 7-*strongly agree*. Three items are reverse-coded. In the first step, the scale was translated with the use of the back-translation procedure. The two translators translated items independently into Polish, then I created the final scale based on these translations; next, the Polish version was translated back into English by the third translator. This back-translated version was compared with the original scale. As the two versions were assessed as being highly similar, the Polish translation was evaluated as being adequate and used in the presented study. The factor structure of the scale was evaluated with the use of CFA. It turned out that the three reverse-coded items had small factor loadings, which is why they were excluded from the analysis. The remaining 23 items of the DAPS created three scales, in line with the questionnaire authors’ assumptions, *X*^2^ (*N* = 459, *df* = 224) = 777.73, *p* < .001, CFI = .91, RMSEA = .07, SRMR = .09, and were used in further analyses. The reliabilities of the subscales were .86, .84, .95, respectively.

#### Children-oriented perfectionism

In order to measure children-oriented perfectionism, the *Children Dyadic Almost Perfect Scale* (C-DAPS), developed by the author of the article, was applied. The original items from the DAPS were modified in such a way as to refer to children and not a partner. Similarly to the original DAPS, the C-DAPS was also composed of 26 items, assessed on a seven-point Likert scale, creating three subscales: High Standards (6 items, e.g. *I have a strong need for my child/children to strive for excellence*), Order (4 items, e.g. *I think my child/children should be organized*), Discrepancy (16 items, e.g. *My children/child often do(es) not measure up to my expectations*). The scale was applied in this form in the reported study. The psychometric parameters of the measure are presented in the Results section. The scale was prepared with a view to parents of children aged at least three years old, because in the case of younger children some items might have turned out to be not suitably adjusted to the child’s age. For the English-language version of the C-DAPS see S1 Appendix; for the Polish version see S2 Appendix.

#### Intimate relationship difficulties

To measure difficulties in an intimate relationship, two short scales were applied. The scales enabled the measurement of two aspects: (1) *Relationship stress*: a short scale based on a similar tool used in examining parental stress was prepared [30, 31]. The scale consists of three items: *The relationship with my partner brings about many more problems than I expected; Being in my current romantic relationship is harder than I thought it would be; Being in my current romantic relationship often makes me feel anxious*. Participants answered on a five-point Likert scale ranging from 1 (*I strongly disagree*) to 5 (*I strongly agree*). In the present study, the Cronbach’s alpha was .91; (2) *Relationship conflicts*: a short scale composed of three items was applied: *My partner regularly scolds me and grumbles; I have the impression that my partner does not understand me; I often argue with my partner*. Subjects responded to the statements using a five-point Likert scale, ranging from 1 (*I strongly* disagree) to 5 (*I strongly agree*). In the presented article, the Cronbach’s alpha of this indicator was .87.

#### Parenting difficulties

Two variables were measured: (1) *Childrearing stress*, which was measured with the use of a scale prepared on the basis of previous studies [30, 31]. The scale was composed of three items that pertained to the perception of childrearing as burdensome and problematic: *Raising my child/children brings about many more problems than I expected; Raising my child/children is harder than I thought it would be; Raising my child/children frequently causes problems*. Items were assessed on a Likert scale ranging from 1 (*definitely disagree*) to 5 (*definitely agree*). The Cronbach’s alpha was .81; (2) *Parental role restrictions*, which was measured with the use of a scale that expressed the degree to which the parent perceives parenthood as limiting and preventing him/her from the lifestyle s/he would like to have. The scale was composed of four items (e.g. *Raising my children prevents me from doing things that are important to me* [30, 31]). Items were assessed on a five-point Likert scale ranging from 1 (*definitely disagree*) to 5 (*definitely agree*). The Cronbach’s alpha was .88.

### Analytic strategy

The analysis was preceded by an evaluation of the factor structure of the Polish version of the C-DAPS as it was the first use of this scale. Confirmatory factor analysis, assessing the fit of the three-factor solution, was applied. Because of the deviation of the multivariate distribution from the normal distribution, the Sattora-Bentler scaled chi-square test [32] was used, a lower value of which indicates a better model fit. The evaluation of the structure of the C-DAPS was conducted on the entire sample (*N* = 459), and separately in the groups of women and men. Three indices of model fit were applied [33]: comparative fit index (CFI), the value of which should be higher than 0.95 (less strict rule of thumb is .90); root mean square error of approximation (RMSEA), the value of which should be lower than .06 (or 0.08 when we use the less strict approach); and standardized root mean square residual (SRMR), the value of which should not be higher than 0.08. Measurement invariance between women and men was also analyzed to determine whether the structure of the C-DAPS is similar across gender. In order to determine invariance, gradually stricter equality constraints were tested [34]: configural invariance (equality of the factor structure), metric invariance (equality of the factor loadings), and scalar invariance (equality of the item intercepts). The factor structure and the measurement invariance were investigated with the use of Mplus 7.3. Subsequently, Pearson’s *r* correlations between all the analyzed variables were performed separately for women and men. In the next step, the differences between women and men in respect of the analyzed variables, using MANOVA analysis, were assessed. Next, within-subject comparisons between partner-oriented perfectionism and child-oriented perfectionism among women and men were conducted, in order to evaluate whether the participants had different expectations towards the partner and the child. For this purpose, repeated-measures MANOVA was applied. Subsequently, multivariate regression analysis was conducted in order to identify specific relationships between the analyzed variables. In the first step, a few controlled variables, including narcissism, were introduced to the model (narcissism was controlled for as it was seen as important to assessing whether child-oriented perfectionism enables explanation of additional variance beyond the earlier reported links); in the second step, the three dimensions of partner-oriented perfectionism were added, and in the third step the three dimensions of child-oriented perfectionism were introduced into the model in order to assess whether this construct enables explanation of an additional variance in the difficulties in the areas of the romantic and the parental relation. In the last step, the interaction terms between gender and perfectionism were included in order to analyze whether gender moderated the analyzed relationships. Four regression models were tested; criterion variables were relationship stress, relationship conflicts, childrearing stress, and parental role restrictions. The analysis of correlations, mean differences and regression was conducted with the use of SPSS 25.

## Results

### The Children Dyadic Almost Perfect Scale (C-DAPS): Psychometric properties in a Polish sample

The CFA conducted on the entire sample showed that the assumed three-factor model of the C-DAPS was not sufficiently fitted to the data, SBS-*X*^2^ (*N* = 459, *d f* = 296) = 1176.10, *p* < .001, CFI = .85, RMSEA = .08, SRMR = .08. Separate analyses conducted in the groups of women and men also led to analogical conclusions. Admittedly, the obtained results were almost identical to the fit parameters in the original DAPS [13]; nonetheless, it was decided to take a closer look at the potential causes of this situation. First of all, it turned out that items: 3, 16, and 21 (all reverse-coded) from the Discrepancy subscale had small factor loadings and were weakly correlated with the total scale score (*r*s about .20‒.30). After excluding these three items from the analysis the model fit increased, although it was still below the acceptance level. Modification indices showed, however, that two pairs of items should be correlated: items 5 and 25 from the Standards subscale, and 10 and 26 from the Discrepancy subscale. After applying these changes, the fit parameters rose to the acceptable level, SBS-*X*^2^ (*N* = 459, *df* = 225) = 770.61, *p* < .001, CFI = .90, RMSEA = .07, SRMR = .08. Separate analyses conducted among women and men led to identical conclusions (women: CFI = .90, RMSEA = .06, SRMR = .07; men: CFI = .91, RMSEA = .07, SRMR = .08). Admittedly, residual correlations break certain assumptions of CFA, yet taking into consideration the fact that the fit of the original version of the DAPS was also below the level usually deemed sufficient [13], it was hard to expect that the modified version would turn out to be much better fitted. On the other hand, the introduced changes were slight. All correlated items came from the same subscales and were similar in respect of their linguistic form. Taking into consideration the result of the CFA, items 3, 16, and 21 were excluded from the analysis similarly to the case of the DAPS. The values of the factor loadings for the remaining 23 items in the entire sample varied between .47 and .88. Cronbach’s alpha for the version composed of 23 items turned out to be high: Standards .86, Order .82, Discrepancy .94. The results of the measurement invariance between women and men are presented in Table 1. Configural invariance was confirmed; however, when additional constraints were added (metric and scalar invariance) the full invariance was not obtained as indicated by ΔCFI (a value higher than .01, in this case .017, indicates that the metric model was significantly less fitted than the configural model) and ΔSRMR (a value higher than .03, in this case .034, indicates that the metric model was significantly less fitted than the configural model). However, it should be noted that the changes in CFI and SRMR were only slightly above the acceptance levels. In the next step, the partial invariance was tested. Modification indices suggested (values over 20) that the three factor loadings (items 1, 12, and 26) and the four item intercepts (items 10, 11, 12, and 22) should be released from being equal. When these modifications were implemented, the differences between the configural, metric, and scalar models indicated acceptable partial invariance across gender (ΔCFI < .01, ΔRMSEA < 0.15, ΔSRMR < 0.03).

**Table 1:** Measurement invariance across gender.

Full invariance	CFI	RMSEA [90%CI]	SRMR
Model 1: Configural invariance	.918	.061	.072
Model 2: Metric invariance	.890 ΔCFI = .017	.066 ΔRMSEA = .004	.106 ΔSRMR = .034
Model 3: Scalar invariance	.873 ΔCFI = .017	.073 ΔRMSEA = .004	.113 ΔSRMR = .007
Partial invariance			
Model 2: Metric invariance	.898 ΔCFI = .009	.067 ΔRMSEA = .002	.091 ΔSRMR = .019
Model 3: Scalar invariance	.892 ΔCFI = .006	.068 ΔRMSEA = .001	.093 ΔSRMR = .002

Partial metric invariance was assessed with items 1, 12, and 26 released from being equal between women and men; partial scalar invariance was assessed with intercepts of items 10, 11, 12, and 22 released from being equal between women and men.

In order to assess whether children-oriented perfectionism could be considered an additional, besides partner-oriented perfectionism, aspect of perfectionism, it was decided to evaluate the fit of two additional models: (1) a three-factor model in which items of the corresponding subscales of the DAPS (23 items) and the C-DAPS (23 items) loaded the same factor (error variances of individual items were not allowed to correlate): Global High Standards (items from the DAPS and the C-DAPS), Global Order, and Global Discrepancy; and (2) a six-factor model in which the subscales of the DAPS and the C-DAPS constituted correlated but independent factors (error variances of individual items were not allowed to correlate). In the case of the global three-factor model, fit was very poor: SBS-*X*^2^ (*N* = 459, *df* = 979) = 4710.84, *p* < .001, CFI = .71, RMSEA = .09, SRMR = .11, whereas in the case of the six-factor model, although characterized by a model fit slightly below the values usually considered to be acceptable, SBS-*X*^2^ (*N* = 459, *df* = 967) = 2499.73, *p* < .001, CFI = .88, RMSEA = .06, SRMR = .08, it was much better fitted than the model with three global factors. We can acknowledge, therefore, that partner-oriented and child-oriented perfectionism are constructs that are associated with each other, but are quite separate.

### Correlations between analyzed variables

In Table 2, the correlations between High Standards, Order, and Discrepancy in the romantic and the parental domain are presented. In the group of women, all subscales of the two questionnaires, the DAPS and the C-DAPS, were positively correlated. Among men, these relationships were similar, except for two differences. In the DAPS, Order and Discrepancy were not correlated, whereas in the C-DAPS, Order and Discrepancy were negatively correlated.

**Table 2:** Correlations between the analyzed variables in women and men.

	1	2	3	4	5	6	7	8	9	10	11
1. Pathological narcissism	-	.34***	.13*	.36***	.28***	.13*	.37***	.37***	.30***	.34***	.30***
2. DAPS Standards	.30***	-	.54***	.59***	.67***	.43***	.42***	.35***	.36***	.14*	.19**
3. DAPS Order	.02	.50***	-	.28***	.42***	.71***	.22***	.16*	.13*	.01	.01
4. DAPS Discrepancy	.47***	.47***	-.06	-	.32***	.17**	.47***	.59***	.60***	.21***	.27***
5. C-DAPS Standards	.37***	.60***	.32***	.33***	-	.59***	.66***	.20**	.22***	.09	.13*
6. C-DAPS Order	-.05	.12	.59***	-.30***	.25***	-	.36***	.13*	.15*	-.01	-.01
7. C-DAPS Discrepancy	.50***	.34***	-.10	.67***	.48***	-.28***	-	.29***	.31***	.28***	.30***
8. Relationship stress	.36***	.45***	.09	.76***	.30***	-.21***	.49***	-	.73***	.25***	.25***
9. Relationship conflicts	.32***	.40***	.05	.73***	.24***	-.23***	.45***	.86***	-	.16***	.24***
10. Childrearing stress	.21**	.16***	.11	.34***	.12	.01	.28***	.44***	.35***	-	.41***
11. Parental role restrictions	.40***	.31***	-.06	.57***	.29***	-.17***	.55***	.56***	.48***	.51***	-

Values for women have been provided above the diagonal, and for men below the diagonal.

* *p* < .05

** *p* < .01

*** *p* < .001

In women and men, the level of narcissism correlated positively with Standards and Discrepancy, both in the romantic and the parental perfectionism domains. In the group of women, narcissism turned out to be positively, although weakly, correlated with Order in the two domains, while in the group of men there was no correlation between narcissism and Order.

The results from the DAPS Standards and the DAPS Discrepancy were more strongly positively correlated with difficulties in the romantic relationship than in the parental relation, both in women and in men. The results of the DAPS Order were very weakly, or were not at all, connected with difficulties in either of these domains. In the case of the subscales of the C-DAPS, it turned out that they correlated weakly with difficulties in the romantic relation and correlated in a similar way to the dimensions of the DAPS with difficulties in the parental relation. The results among women and men were similar in this respect.

Additionally, correlations between the analyzed variables and the age of the participants, the age of their children, and the number of children they had were assessed. In the vast majority of cases, the correlations were insignificant. It turned out, however, that with age the level of parents’ narcissism tended to be lower (*r* = -.22 in women and *r* = -.16 in men); the older the children were, the lower was the level of conflicts with the partner (*r* = -.12) and parental role restrictions among women (*r* = -.16) and parental stress among men (*r* = -.15). In the group of women, the number of children was also negatively correlated with the results on the DAPS High Standards scale (*r* = -.14) and the DAPS Order scale (*r* = -.14).

### Gender differences

In the next step, women and men were compared in respect of the analyzed variables: narcissism and the three dimensions of partner-oriented perfectionism and the three dimensions of child-oriented perfectionism. A significant, relatively strong multivariate effect of gender was observed: Wilks’ *λ* = .73, *F* (11, 447) = 14.82, *p* < .001, *η*^2^ = .27. The results of the univariate analysis are presented in Table 3. Men obtained significantly higher results in respect of narcissism, the C-DAPS Standards, the DAPS and C-DAPS Discrepancy, relationship conflicts, and parental role restrictions. Women, in turn, were characterized by a higher level of the DAPS Order and childrearing stress. There were no gender differences in respect of the DAPS Standards, the C-DAPS Order, and stress in intimate relationships.

**Table 3:** Differences between women and men in respect of the analyzed variables.

	Women		Men		*F*, *η*^2^
	*M*	*SD*	*M*	*SD*	
1. Pathological narcissism	1.99	.82	2.19	.90	6.48*, .01
2. DAPS Standards	3.75	1.22	3.93	1.06	*ns*
3. DAPS Order	5.32	1.02	4.77	1.08	30.80***, .06
4. DAPS Discrepancy	2.85	1.03	3.30	1.07	20.58***, .04
5. C-DAPS Standards	3.21	1.22	3.88	.94	40.77***, .08
6. C-DAPS Order	5.06	1.03	4.98	.94	*ns*
7. C-DAPS Discrepancy	2.15	.79	2.98	1.17	82.53***, .15
8. Relationship stress	2.36	1.23	2.57	1.13	*ns*
9. Relationship conflicts	2.35	1.11	2.64	1.04	7.58**, .02
10. Childrearing stress	3.33	1.01	3.14	.91	4.34*, .01
11. Parental role restrictions	2.23	.95	2.61	.98	16.61***, .04

* *p* < .05

** *p* < .01

*** *p* < .001

### Within-subject mean comparisons

In order to verify whether the participants differed in respect of partner-oriented and child-oriented perfectionism, a repeated-measures MANOVA was conducted. The results showed significant within-subject multivariate effects among women, Wilks’ *λ* = .64, *F* (3, 261) = 48.71, *p* < .001, *η*^2^ = .36, and men, Wilks’ *λ* = .84, *F* (3, 192) = 14.82, *p* < .001, *η*^2^ = .16. The univariate comparisons are displayed in Table 4. Among women, in the case of each pair of variables, significantly higher results were obtained in the romantic domain. The strongest effect could be observed in the case of the Discrepancy subscale. Among men, differences in perfectionism in the two domains were smaller and not always the same as among women. First of all, High Standards in the romantic and the parental domain did not differ from each other. Secondly, men obtained a higher result on the Order scale in the parental domain. When it comes to Discrepancy, in men, as in women, a higher result was observed in the romantic domain.

**Table 4:** Within-subject comparisons between partner dyadic perfectionism and children dyadic perfectionism.

	Women			Men		
	*M*_1_ –*M*_2_	*F*	*η*^2^	*M*_1_ –*M*_2_	*F*	*η*^2^
Partner High Standards–Child High Standards	.54	78.44***	.23	.06	.81 *ns*	< .01
Partner Order–Children Order	.26	30.03***	.10	- .20	9.48**	.05
Partner Discrepancy–Children Discrepancy	.70	140.52***	.35	.33	24.45***	.11

* *p* < .05

** *p* < .01

*** *p* < .001

### Narcissism, partner-oriented perfectionism, and child-oriented perfectionism as specific predictors of difficulties in the romantic and parental domains

The next step was the analysis of specific relationships between narcissism, partner-oriented perfectionism, and child-oriented perfectionism, and difficulties in the romantic and the parental relations. For this purpose, four separate regression models were conducted. Considering the weak but significant relationships between some of the analyzed variables and the participants’ age and the age and number of their children, and significant gender differences, these variables were controlled for by introducing them into the regression models in the first step along with narcissism. In the second step, the three dimensions of the DAPS, and in the third step the three dimensions of the C-DAPS were added, and in the fourth step six interaction terms (gender and the six perfectionism dimensions) were introduced. The criterion variables were, respectively: relationship stress, relationship conflicts, childrearing stress, and parental role restrictions. All VIF values were below 4, suggesting that multicollinearity did not strongly affect the results. The results of the regression analyses in the form of standardized beta values are presented in Table 5.

**Table 5:** The results of regression analyses.

	Relationship stress	Relationship conflicts	Childrearing stress	Parental role restrictions
Step 1				
Sex	.05 (.11)	.10* (.10)	-.11* (.09)	.17* (.09)
Age	-.01 (.02)	.01 (.01)	.06 (.01)	.05 (.01)
Child’s/childrens’ age	.05 (.02)	-.03 (.02)	-.11* (.02)	-.13** (.02)
Number of children	-.01 (.08)	-.04 (.08)	-.01 (.07)	.03 (.07)
Narcissism	.36*** (.06)	.30*** (.06)	.29*** (.05)	.34*** (.05)
*R*^2^	.14	.11	.10	.17
*F*	14.01***	10.49***	9.73***	18.02***
Step 2				
	-.03 (.09)	-.01 (.09)	-.13** (.10)	.09 (.09)
Age	.01 (.01)	.02 (.01)	.06 (.01)	.06 (.01)
Child’s/childrens’ age	.06 (.02)	-.04 (.01)	-.10 (.02)	-.14** (.01)
Number of children	.02 (.07)	-.02 (.06)	.01 (.07)	.05 (.06)
Narcissism	.12** (.06)	.03 (.05)	.21*** (.06)	.20*** (.05)
DAPS Standards	-.02 (.06)	.01 (.05)	-.06 (.06)	.05 (.05)
DAPS Order	.03 (.05)	-02 (.05)	.10 (.05)	-.13* (.05)
DAPS Discrepancy	.62*** (.05)	.64*** (.05)	.23*** (.05)	.32*** (.05)
*R*^2^ (Δ*R*^2^)	.44 (Δ*R*^2^ = .30***)	.43 (Δ*R*^2^ = .32***)	.13 (Δ*R*^2^ = .03**)	.26 (Δ*R*^2^ = .09***)
*F*	43.19***	41.87***	8.10***	19.50***
Step 3				
Sex	-.07 (.10)	-.01 (.10)	-.17** (.06)	.03 (.10)
Age	.01 (.01)	.02 (.01)	.06 (.01)	.07 (.01)
Child’s/childrens’ age	.07 (.02)	-.04 (.01)	-.11* (.02)	-.16** (.01)
Number of children	.03 (.07)	-.02 (.06)	.01 (.07)	.04 (.06)
Narcissism	.14** (.06)	.04 (.06)	.18*** (.06)	.15** (.06)
DAPS Standards	.01 (.07)	.01 (.06)	-.01 (.07)	.07 (.06)
DAPS Order	.04 (.07)	-.05 (.06)	.02 (.07)	-.08 (.06)
DAPS Discrepancy	.65*** (.06)	.65*** (.05)	.13 (.06)	.18** (.05)
C-DAPS Standards	-.01 (.07)	-.02 (.06)	-.11 (.07)	-.06 (.06)
C-DAPS Order	-.04 (.07)	.04 (.06)	.02 (.06)	-.02 (.06)
C-DAPS Discrepancy	-.08 (.07)	-.01 (.07)	.21** (.07)	.29*** (.07)
*R*^2^ (Δ*R*^2^)	.45 (Δ*R*^2^ < .01)	.43 (Δ*R*^2^ < .01)	.15 (Δ*R*^2^ = .02*)	30 (Δ*R*^2^ = .04***)
*F*	31.68***	30.36***	6.64***	16.88***
Step 4				
Sex*DAPS Standards				
Sex*DAPS Order		.20* (.08)		
Sex*DAPS Discrepancy			.20* (.14)	.17* (.14)
Sex* C-DAPS Standards				
Sex* C-DAPS Order		-.20** (.13)		
Sex* C-DAPS Discrepancy			-.29* (.14)	
*R*^2^ (Δ*R*^2^)	.46 (Δ*R*^2^ = .01)	.45 (Δ*R*^2^ = .02*)	.19 (Δ*R*^2^ = .04**)	.30 (Δ*R*^2^ = .01)
*F*	21.52***		5.78***	11.29***

* *p* < .05

** *p* < .01

*** *p* < .001

The table presents standardized beta values obtained, successively, in each of the four regression models; standard errors are displayed in parentheses.

Among the variables controlled for in the first step, narcissism was the strongest predictor of relationships stress, childrearing stress, and parental role restrictions. Several weak relationships were observed for gender, as was reported earlier, and children’s age (parental stress and role restrictions were higher when the child was younger).

When the DAPS dimensions were introduced in the second step, in all four regression models, the relationship between narcissism and the criterion variables decreased, especially in the cases of the two partner-related variables. In the case of relationship conflicts the link with narcissism even became insignificant. In general, these observations suggest that partner-oriented perfectionism could mediate, partially or fully, the influence of narcissism on relational and parental difficulties. While DAPS high standards and order were not significantly related to any of the criterion variables, DAPS discrepancy was positively and significantly related to all of them. However, the links with the two relationship-related variables (relationship stress and relationship conflicts) were strong (betas .62‒.64), while the links with the parenthood-related difficulties turned out to be small to moderate (betas .22‒.32).

When the dimensions of child-oriented perfectionism were introduced in the third step, it turned out that they were not related to romantic relationship difficulties and did not explain an additional part of the variance of these variables. However, the results were different for parental difficulties. In the case of childrearing stress, its relationship with DAPS discrepancy became insignificant, and only C-DAPS discrepancy proved to be a significant predictor of this variable among perfectionism dimensions. Adding child-oriented perfectionism also enabled the additional variance in the criterion variable to be explained. In the case of parental role restrictions, the results were similar, except the link with DAPS discrepancy remained significant, albeit diminished. In both the cases of childrearing stress and parental role restrictions, child-oriented discrepancy was the strongest predictor among those analyzed. The relationships between child-oriented perfectionism and parental difficulties were small to moderate (betas .21‒.29), and the variance explained by all predictors was 15%‒30%. As regards high standards and order, both in the partner and children domains, it turned out that despite their significant bivariate correlations with the criterion variables, main effects of these factors were not present when the other dimensions were controlled for.

As a correlational analysis and a gender difference analysis showed that women and men differed in respect of the analyzed constructs, gender was included in the regression analysis as a potential moderator of the relationships between perfectionism and the difficulties in relational and parental functioning. In Table 5, the interaction terms that turned out to be significant are presented. Subsequent analyses conducted in order to understand these interactions showed that several aspects of partner- and child-oriented perfectionism could differently influence the functioning of women and men. While expecting order from a partner or a child was not a significant predictor of the criterion variables in the whole sample (step 3), it turned out that it was related to relationship conflicts in the subsample of women. Interestingly, expecting order from a partner (DAPS Order) was associated with a lower level of relationship conflicts (*β* = -.18, *p* < .05), but expecting order from a child was related to a higher intensity of conflicts with a partner (*β* = .19, *p* < .05). Also, partner-oriented and child-oriented discrepancy seem to have a different meaning for women and men. Childrearing stress in women was not related to partner-oriented discrepancy at all, but in men this relationship was positive and quite strong (*β* = .42, *p* < .05). Conversely, childrearing stress in women was much more strongly related to child-oriented discrepancy (*β* = .32, *p* < .001) than in men (*β* = .13, *ns*). As regards parental role restrictions, the results showed that their positive association with partner-oriented discrepancy observed in step 3 was significant only for men (*β* = .38, *p* < .001). In sum, the interaction analysis added several significant observations. While the level of partner-oriented and child-oriented perfectionism in parents, in general, influenced their functioning in a similar way (e.g. discrepancy is related to more familial difficulties), we also observed that social and emotional effects of other-oriented perfectionism in a family context are gender-specific to some extent.

Finally, it should be noted that the analyzed predictors explained more variance of the relational variables (relationship stress: 46%, and relationship conflicts: 45%) than the parental variables (childrearing stress: 19%, and parental role restrictions: 30%).

## Discussion

The aim of the reported study was to deepen the knowledge about other-oriented perfectionism in concrete, close relationships. It was verified whether partner-oriented perfectionism and child-oriented perfectionism are connected with each other and whether it can be claimed that perfectionism in each of these domains has at its base in narcissistic needs. The differences in the intensity of perfectionism in both domains were also analyzed. Finally, an attempt was made to evaluate whether partner-oriented perfectionism and child-oriented perfectionism can be considered specific predictors of difficulties in the romantic and in the parental domain and whether these relationships are related to gender. As the present study was the first to directly evaluate child-oriented perfectionism in parents, it required an appropriate measure to be prepared. In order to achieve this aim, the Children Dyadic Almost Perfect Scale (C-DAPS; see Appendix) was created based on a well-known scale by Shea, Slaney, and Rice [13]. The obtained results suggest that the C-DAPS is a valid and reliable measure with good psychometric parameters comparable with the original DAPS scale. The hypothesized factor structure and gender invariance were only acceptably fitted; however, taking all results into account, the C-DAPS can be assessed positively.

In line with the predictions formulated in the first hypothesis, it turned out that partner-oriented and child-oriented perfectionism are positively connected with each other. The moderate strength of the relationship between these two aspects of perfectionism and the results of confirmatory factor analysis suggest, however, that these constructs are, at least partially, independent of each other and that one can expect the occurrence of individual differences in respect of whether perfectionistic expectations are directed at the child or the partner. It would be worth exploring this thread further in future studies. Simultaneously, the positive relationship of partner- and child-oriented perfectionism with narcissism indicates that both of these aspects of other-oriented perfectionism can have a shared basis, i.e. narcissistic motivation to protect and strengthen the person’s unstable self-esteem [5].

Mitchelson and Burns [24] and Dunn, Dunn, and McDonald [25] demonstrated that general other-oriented perfectionism can have different levels in different domains, e.g. in work and in family. As regards the second hypothesis, the results suggest that in specific domains, such as a family domain as analyzed here, people also have different expectations for particular people. Among women, perfectionistic standards and order, and also perfectionistic discrepancy, pertain to a greater extent to the partner than to the child/children. The strongest effect is observed in the case of discrepancy, suggesting that women far more frequently have a sense that it is the partner who does not live up to their expectations, not the child/children. An analogical result can be observed in the case of men. It suggests that people in general tend to accept to a greater extent the mistakes and ‘imperfection’ of their children than those of their partner. Dunn, Dunn, and McDonald [25] explain that general other-oriented perfectionism is stronger in those domains to which the person has assigned a higher value. Interpreting the obtained results from this perspective, it can be suggested that the participants had higher perfectionistic attitudes towards the partner than the child/children because it is the everyday interactions with their partner that are more strongly associated with their self-esteem. Thus, in order to strengthen and protect it they formulate strong perfectionistic expectations, wanting to ‘adjust’ the partner to their narcissistic fantasies. The observed differences between women and men pertained, in turn, to expecting order and organization (Order). The women tended to expect it more often from the partner, whereas the men expected it from their children, which can also be culturally conditioned and be considered a relic of the traditional organization of family life. In general, the obtained results suggest that narcissistic perfectionists can ‘choose’ individuals towards whom they formulate higher expectations than those directed towards other people, even within the same domain. The results of the study do not enable us, however, to give an answer to the question about the mechanism on which this process is based and why expectations pertain to a greater extent to the partner than to the child. It is an issue worth exploring in future studies.

The most important area, and an element of the third hypothesis, was specific relationships between partner- and child-oriented perfectionism, and difficulties in the spheres of the romantic and the parental relation. The reported study confirmed earlier observations [13] that the role of high standards and order in predicting difficulties in a romantic relationship is small. The intensification of high standards is sometimes considered to be a healthy aspect of perfectionism [26] that does not have to lead to problems. Since the present study analyzed various difficulties, this view can justify the lack of significant, specific relationships with this dimension of perfectionism.

It turned out that a decisive factor for perceived difficulties in the romantic and in the parental domain was, in the first place, the level of discrepancy, which is a finding that remains consistent with findings reported earlier [13]. In line with what had been assumed, it turned out that a large discrepancy between expectations and the degree of their fulfillment by the partner is associated with difficulties in the romantic relation, i.e. it can lead to conflicts and cause the relationship to become a source of stress. At the same time, the connection of partner-oriented perfectionism with difficulties in the parental relation was much weaker or insignificant, especially when child-oriented perfectionism was controlled for in regression analysis. In turn, child-oriented discrepancy is linked to difficulties in the parental relation such as the level of childrearing stress and parental role restrictions, and it seems that it does not have a strong, specific effect on difficulties in the romantic domain.

In several cases, interaction between gender and partner- and child-oriented perfectionism was also observed. This finding indicates that partner-oriented perfectionism can have a stronger influence on men’s parental functioning and that it spilled over into the parental domain more than in the case of women. As regards child-oriented perfectionism, its spillover into relational difficulties was much smaller. When men feel that their partner does not fulfill their expectations (DAPS discrepancy) they also feel more parental stress and perceive parenting as more strongly restricting their autonomy. However, in women this effect was not observed. Conversely, it turned out that for women child-oriented discrepancy more strongly impacted their parental stress than was the case for men. These observations may indicate that other-oriented perfectionism may influence differently the roles that women and men play in a family. Women are expected to take primary care of their children, which makes them more vulnerable to parental stress [35, 36]. This was also observed in the study sample. As a result, when a woman finds that her child does not meet her expectations she might perceive it as more stressful than a man. As regards men, the obtained results are also in accordance with a previously reported spillover effect in a family context [37]. As the fathering role is less well defined it is more determined by the marital relationship than in the case of the mother’s role [37]. As a result, when the relationship satisfaction is low it influences fathering more strongly than mothering. It seems that this effect also occurred in the present study. Men who perceive their partners as not fulfilling expectations, which results in higher relational stress and conflicts, experience in parallel a decrease in their parental adjustment. Further, as they are not usually the main caregivers and the parental role is less salient for them [35, 36], a higher child-oriented perfectionism does not translate to parental stress as strongly as in the case of women.

The obtained results provide a rationale for further studies on child-oriented perfectionism in parents, indicating that it is a construct that can enable perfectionism researchers to attain additional information that is inaccessible with the use of the general other-oriented perfectionism approach. The observations also constitute a support for the thesis about the domain specificity of other-oriented perfectionism [21–23]. However, it turned out that perfectionism can have some specificity even *within a domain*. Perfectionists can formulate differently their expectations towards individuals who belong to the same domain, for instance the family domain, which can be conducive to experiencing specific difficulties in a particular relation. Child-oriented perfectionism constitutes a specific predictor of the quality of the parental relation and as such it should be treated as an additional source of knowledge about the parents as well as giving information about other manifestations of perfectionism. The obtained results may also be significant for further studies on determinants of the development of perfectionism in children and adolescents. Experiences acquired in the family of origin are crucial for the development of perfectionism, particularly the parents’ criticism, their authoritarian attitude towards the child, the lack of permission to make mistakes, and dysfunctional psychological control [1, 2, 19, 20, 38]. The parents’ perfectionism is also inter-generationally transferred on to children. It has been demonstrated, for instance, that general other-oriented perfectionism of the parents’ is associated with a higher level of standards and perfectionistic concerns in the child [39]. Until now researchers of perfectionism have not had at their disposal a scale that would enable them to measure child-oriented perfectionism and that could be used in the exploration of this specific form of perfectionism in the context of close caring and rearing relations from the perspective of the perfectionistic parent. Such a scale can enable us to better understand both the mechanisms of intergenerational transfer of perfectionism and the functioning of perfectionists in the context of the family and to supplement our knowledge derived from previous studies focused on retrospective assessment of parental perfectionism [1]. Wang [40] observes that individuals who think that members of their family expect perfection from them (family-prescribed perfectionism) have lower self-esteem and higher levels of depression symptoms. Child-oriented perfectionism can be useful in explaining these types of observations by looking at them from the other side, i.e. the perspective of the parent. Studies have shown that perfectionism in parents is positively related to child distress [41] and that narcissistic parents more frequently use negative parenting methods, including violence [42]. A study conducted by Piotrowski [27] demonstrates, in turn, that mothers who expect perfection from others can also more often regret becoming a parent and show lower commitment to the parental role. This, going further, can open the way to maladaptive behaviors towards the child. It is suggested here that including, in studies on perfectionism, specific perfectionistic expectations towards children can enrich significantly our understanding of the role of perfectionism in families, both from the point of view of perfectionistic parents and their children.

### Limitations of the study

Although the present study yields new knowledge, the results obtained have to be analyzed in the context of certain limitations. First of all, the presented results come from a cross-sectional study. In particular, the relationships between perfectionism in different domains and difficulties in the romantic and in the parental sphere need to be verified in a longitudinal study. Secondly, the reported study was the first one to measure perfectionism oriented specifically at children, which makes it necessary to replicate it and verify its results. Thirdly, perfectionism and familial difficulties were measured only with the use of the questionnaire method and only from the perspective of the investigated individual. As a result, it was not possible to observe a dyadic interaction and a possible mutual relationship between partner-oriented and child-oriented perfectionism in couples. Further research should assess this dyadic aspect more deeply, with different sources of information and both self-report and objective indicators of perfectionism. The results also need to be verified in studies that will involve more family members, for example parents and children. Fourthly, the study only investigated pathological narcissism at the general level, without differentiating between its grandiose and vulnerable aspect, which would be worth doing in future studies. As the study was conducted in Poland, a central European country, there is a need to replicate it in other cultural contexts, as it could limit the generalizability of the findings. Fifthly, the study used a convenience sample that might have an effect on the validity and generalizability of the results. Finally, the statistical parameters of the C-DAPS (i.e. factor structure and measurement invariance) were only acceptable and the presented scale seems to possess both advantages and disadvantages of the APS and DAPS [13, 43]. Therefore, in future studies, the psychometric properties of the C-DAPS should be carefully evaluated further, especially in cultures other than Polish. It is also recommended that further studies should be conducted on child-oriented perfectionism in order to assess the reliability and utility of this construct.

## Supporting information

S1 Appendix English language version of the C-DAPS. (DOCX)

S2 Appendix Polish language version of the C-DAPS (used in the present study). (DOCX)
