# Supplementary material for: Child-oriented and partner-oriented perfectionism explain different aspects of family difficulties
Source: PLoS One. 2020 Aug 19;15(8):e0236870. doi: 10.1371/journal.pone.0236870 (PMC7437722; doi:10.1371/journal.pone.0236870)
Supplement: S1 Appendix — (DOCX) [file pone.0236870.s001.docx]

APPENDIX 1. English language version of the C-DAPS

The Children Dyadic Almost Perfect Scale (C-DAPS)

Instruction

The following items are designed to measure attitudes people have about their children. There are no right or wrong answers. Please respond to all of the items. Use your first impression and do not spend too much time on individual items. When responding to the items think about your child/children aged 3 or older. Some of the items may not be applicable to children younger than 3. If you have more than one child think about your general attitudes toward them.

Respond on the answer line to the left of each item by using the scale below to describe your degree of agreement or disagreement with each statement.

| 1  Strongly Disagree | 2  Disagree | 3  Slightly Disagree | 4  Neutral | 5  Slightly Agree | 6  Agree | 7  Strongly Agree |
| --- | --- | --- | --- | --- | --- | --- |

| ____1. | I often feel disappointment after my child/children complete(s) a task because I know  that she/he/they could have done better. |
| --- | --- |
| ____2. | I expect my child/children to be (an) orderly person(s). |
| ____3. | My child/children can generally meet the standards that I have set for him/her/them. |
| ____4. | My child/children rarely live(s) up to my standards. |
| ____5. | I have very high standards for my child/children. |
| ____6. | My child’s/children’s best rarely seems to be enough for me. |
| ____7. | Neatness should be important to my child/children. |
| ____8. | I expect the best from my child/children. |
| ____9. | I am rarely satisfied with my child’s/children’s accomplishments. |
| ____10. | I often feel frustrated because my child/children do(es) not meet the goals I have for him/her/them. |
| ____11. | I expect my child/children to try to do her/his/their best at everything she/he/they do(es). |
| ____12. | I have trouble with my child/children leaving things incomplete. |
| ____13. | My child’s/children’s best never seems to be good enough for me. |
| ____14. | I have high standards for my child’s/children’s performance at preschool or at school. |
| ____15. | My child/children often do(es) not measure up to my expectations. |
| ____16. | I usually feel like what my child/children has/have done is good enough. |
| ____17. | I think my child/children should be organized. |
| ____18. | I am hardly ever satisfied with my child’s/children’s performance. |
| ____19. | I have a strong need for my child/children to strive for excellence. |
| ____20. | My child/children is/are seldom able to meet my standards for performance. |
| ____21. | I usually feel pretty satisfied with what my child/children do(es). |
| ____22. | I expect my child/children to think things should be put away in their place. |
| ____23. | My child’s/children’s performance rarely measures up to my standards. |
| ____24. | I am not satisfied, even when I know my child/children has/have done his/her/their best. |
| ____25. | I have high expectations of my child/children. |
| ____26. | I can get pretty upset when my child/children doesn’t/don’t do as well as I think she/he/they should. |

SCORING FOR CHILDREN DYADIC APS:

*High Standards*: 5, 8, 11, 14, 19, 25

*Order*: 2, 7, 17, 22

*Discrepancy*: 1, (3), 4, 6, 9, 10, 12, 13, 15, (16), 18, 20, (21), 23, 24, 26

NOTE: Items 3, 16, and 21 from the Discrepancy subscale (all are reverse coded) were excluded from the analysis. However, using them in other samples is not precluded but in such a case checking their psychometric parameters is highly recommended.
