# Supplementary material for: Child-oriented and partner-oriented perfectionism explain different aspects of family difficulties
Source: PLoS One. 2020 Aug 19;15(8):e0236870. doi: 10.1371/journal.pone.0236870 (PMC7437722; doi:10.1371/journal.pone.0236870)
Supplement: S2 Appendix — (DOCX) [file pone.0236870.s002.docx]

Appendix 2. Polish language version of the C-DAPS (used in the present study)

The Children Dyadic Almost Perfect Scale (C-DAPS)

Instrukcja

Poniższe stwierdzenia służą do oceny tego jakie uczucia towarzyszą ludziom wychowującym dzieci. Nie ma tutaj dobrych lub złych odpowiedzi. Proszę, oceń wszystkie stwierdzenia. Oprzyj się na swoim pierwszym wrażeniu i nie spędzaj zbyt dużo czasu nad pojedynczymi pozycjami. Udzielając odpowiedzi, myśl o swoim dziecku (dzieciach), które ukończyło(-y) 3 lata. Niektóre z pozycji mogą być niedopasowane do sytuacji młodszych dzieci.

Użyj poniższej skali odpowiedzi. Obok każdego ze stwierdzeń, po lewej stronie, wpisz wartość od 1 do 7, która najlepiej wyraża stopień w jakim zgadzasz się z danym stwierdzeniem.

| 1  Zdecydowanie się nie zgadzam | 2  Nie zgadzam się | 3  Raczej się nie zgadzam | 4  Ani tak, ani nie; trudno powiedzieć | 5  Raczej się zgadzam | 6  Zgadzam się | 7  Zdecydowanie się zgadzam |
| --- | --- | --- | --- | --- | --- | --- |

| ____1. | Często czuję rozczarowanie, kiedy moje dziecko (dzieci) coś robi(-ą), ponieważ wiem że mogłoby (mogłyby) to zrobić lepiej. |
| --- | --- |
| ____2. | Chcę, aby moje dziecko (dzieci) było(-y) uporządkowane i dobrze zorganizowane. |
| ____3. | Moje dziecko (dzieci) najczęściej potrafi(-ą) spełnić wymagania jakie mu (im) stawiam |
| ____4. | Moje dziecko (dzieci) rzadko potrafi(-ą) spełnić moje wymagania. |
| ____5. | Mam bardzo duże wymagania wobec mojego dziecka (moich dzieci). |
| ____6. | Nawet gdy moje dziecko (dzieci) da (dadzą) z siebie wszystko, to rzadko jest to dla mnie wystarczające. |
| ____7. | Czystość i schludność powinny być ważne dla mojego dziecka (moich dzieci). |
| ____8. | Od mojego dziecka (moich dzieci) oczekuję idealnego wykonania tego co robi. |
| ____9. | Rzadko kiedy jestem usatysfakcjonowany/-a kiedy moje dziecko (dzieci) coś zrobi(-ą). |
| ____10. | Często się denerwuję, ponieważ moje dziecko (dzieci) nie robi(-ą) czegoś tak jak bym tego oczekiwał(-a). |
| ____11. | Oczekuję od mojego dziecka (moich dzieci), że robiąc coś, zawsze da(-dzą) z siebie wszystko. |
| ____12. | Mam problem z zaakceptowaniem sytuacji, gdy moje dziecko (dzieci) pozostawia(-ją) jakieś zadania niedokończone. |
| ____13. | Nawet kiedy moje dziecko (dzieci) zrobi(-ą) coś najlepiej jak potrafi(-ą), to nigdy nie jest to dla mnie zrobione wystarczająco dobrze. |
| ____14. | Mam duże wymagania wobec tego, jak moje dziecko (dzieci) powinno (powinny) sobie radzić w przedszkolu lub w szkole. |
| ____15. | Moje dziecko (dzieci) często nie spełnia(-ją) moich oczekiwań. |
| ____16. | Zazwyczaj uważam, że coś co zrobiło(-y) moje dziecko (dzieci), jest zrobione wystarczająco dobrze. |
| ____17. | Uważam, że moje dziecko (dzieci) powinno(-y) być dobrze zorganizowane. |
| ____18. | Prawie nigdy nie jestem zadowolony/-a z efektów tego co zrobi(-ą) moje dziecko (dzieci) |
| ____19. | Chcę, aby moje dziecko (dzieci) starało (-y) się robić wszystko doskonale. |
| ____20. | Moje dziecko (dzieci) rzadko jest (są) w stanie zrobić coś tak dobrze, jak bym tego oczekiwał/-a. |
| ____21. | Zazwyczaj jestem dość zadowolony/-a z tego, co i jak robi (-ą) moje dziecko (dzieci). |
| ____22. | Oczekuję od mojego dziecka (moich dzieci), że będzie (będą) odkładało(-y) rzeczy na swoje miejsce. |
| ____23. | Efekty tego co robi(-ą) moje dziecko (dzieci), rzadko spełniają moje wymagania. |
| ____24. | Nie jestem usatysfakcjonowany/-a nawet wtedy, gdy wiem że moje dziecko (dzieci) dało (-y) z siebie wszystko. |
| ____25. | Mam duże oczekiwania wobec mojego dziecka (moich dzieci). |
| ____26. | Potrafię się naprawdę zdenerwować kiedy moje dziecko (dzieci) nie wykonuje (-ą) czegoś tak dobrze, jak według mnie powinno (powinny). |

KLUCZ:

Wysokie Standardy *(High Standards)*: 5, 8, 11, 14, 19, 25

Porządek *(Order)*: 2, 7, 17, 22

Rozbieżność *(Discrepancy)*: 1, (3), 4, 6, 9, 10, 12, 13, 15, (16), 18, 20, (21), 23, 24, 26

UWAGA: Pozycje 3, 16 i 21 ze skali Rozbieżność (wszystkie te pozycje są odwrócone) zostały wykluczone z analizy. Nie należy brać ich pod uwagę przy obliczaniu wyniku dla skali Rozbieżność.
